# A Preliminary proteomics-based assessment of biotic indicators in Central Mexican water bodies biotic indicators by proteomics in Mexican water bodies

**DOI:** 10.1371/journal.pone.0342705

**Published:** 2026-02-26

**Authors:** Catalina E. Gardella-García, Eduardo Domínguez-de-la-Cruz, Gerardo Pérez-Ramírez, Randy E. David, Juan Enrique Chacón-Hernández, Sandra Cotino-Nájera, María de Lourdes Muñoz

**Affiliations:** 1 Genetics and Molecular Biology Department, Center for Research and Advanced Studies of the National Polytechnic Institute, Instituto Politécnico Nacional Avenue, San Pedro Zacatenco, Mexico City, Mexico; 2 Family Medicine and Public Health Sciences, Wayne State University School of Medicine, 540 E Canfield St, Detroit, Michigan, United States of America; Universidad de Guadalajara, MEXICO

## Abstract

Human activities such as industrialization, recreation, domestic water use, agriculture, and energy production have significantly increased pollution in water bodies, especially within urban regions. This pollution affects ecological biodiversity, exerting broad impacts on various environmental and public health factors. In response, this study utilized proteomic analysis to characterize the macro- and microbiota community compositions of natural water bodies located across diverse urban and semi-urban regions. Our strategy involved collecting samples from Mexico City as well as the Mexican states of Mexico, Morelos and Guanajuato. Samples were analyzed using liquid chromatography, coupled with High Resolution Mass Spectrometry (LC-HDMS), to detect organisms. Using this approach, we identified protein peptide sequences belonging to diverse taxa, including Plantae, Animalia, Protozoa and Bacteria, encompassing both pathogenic and non-pathogenic organisms. Some identified pathogens are known contributors to disease outbreaks in Mexico. Additionally, other detected organisms provided insights into the ecological structure and biodiversity of the regions surrounding each sampled water body. Further analyses included assessments of species richness, relative abundance, and overall biological diversity. Results provide a fundamental proteomic basis for studying biodiversity in Mexican water bodies, which may serve as a reference for future longitudinal research. Moreover, this approach offers a powerful method for detecting and modeling water contamination caused by human activity.

## Introduction

Understanding the processes that influence the biodiversity and functioning of freshwater macro- and microbiota ecosystems is critical for their conservation and sustainable management. Freshwater bodies and their associated ecosystems host diverse biotic communities, including Bacteria, Fungi, Archaea, and viruses, which play an important role in biochemical cycles such as carbon, nitrogen fixation, denitrification, methane production, sulfate reduction, and metal transformation. These are vital processes for maintaining healthy ecosystem balance and function. Certain organisms can be bioindicators of environmental disturbances, signaling pollution from anthropogenic activities. These alterations have been associated with significant economic and public health impact [1–3].

In Mexico, most freshwater bodies are subject to significant contamination, mainly due to a lack of adequate sanitation infrastructure, operational failures in existing treatment facilities, limited application of environmental regulations, and insufficient public policy for wastewater reuse. Climate change, economic demands from industry, and demographic shifts exacerbate these issues [4]. According to data from the Mexican Ministry of Health, water sources in the Valley of Mexico frequently experience periods of scarcity, and are highly contaminated [5]. Between 2000 and 2018 only 35.36% of wastewater in Mexico was treated. Of the treated wastewater, 10%, 82%, and 8% received either primary (adjust pH and remove organic and inorganic materials in suspension with dimensions greater or equal to 0.1 mm), secondary (remove organic and dissolved materials), and tertiary (remove dissolved materials including gases, natural and synthetic organic substances, ions, Bacteria, and viruses) treatments, respectively [6].

Despite these efforts, 54% of untreated wastewater from Mexico City is discharged directly into rivers or streams leading to the accumulation of harmful pollutants such as fecal coliforms, pathogenic organisms, heavy metals, plasticizers, and surfactants [7,8] in Cuernavaca, Morelos the presence of analgesic and anti-inflammatory drugs have also been detected [9]. This situation poses a serious risk to public health interests, causing thousands of deaths annually attributed to diarrheal diseases caused by inadequate water sanitation [10,11]. Between 2007 and 2017, an average of 458,064 new annual cases of acute diarrhea were reported [12].

Research on pathogenic agents in Mexican water bodies has thus far focused on known microbial groups and total coliforms, including *Entamoeba histolytica, Giardia, Salmonella, Shigella, Campylobacter, Yersinia, Vibrio cholerae*, *Escherichia, Helicobacter pylori*, *Leptospira,* and *Rotavirus* [7,13,14]*.*Surface water pollution is particularly pronounced in urban and semi-urban areas, where altered physicochemical conditions negatively impact aquatic habitats and threaten the survival of key species in the trophic chain. This can facilitate the invasion of exotic species, further destabilizing the ecosystem [15,16].Given the vital role of surface waters for human purposes, including for drinking, irrigation, recreation, and industrial use, it is essential to monitor these resources in an effort to mitigate public health risk.

Proteomic-based analyses are powerful tools for characterizing microbial communities and their functional role. In recent years, proteomic methodologies have emerged as valuable instruments in the search for protein biomarkers, that can be used to identify and monitor peptides associated with specific pathogens [17–20]. This study has the goal of applying proteomic approaches to analyze the microbial and non-microbial composition of six urban or semi-urban water bodies within central Mexico. The focus of this study is on identifying protein peptides from pathogenic and/or ecosystem-associated organisms to assess biodiversity and conservation status in these areas. This research will simultaneously identify pathogens and other biotic components linked to human activities that contribute to diarrheal and enteric diseases, as well as ecosystem alterations. Such knowledge is necessary to understand and better manage the complex interactions within microbial and non-microbial communities in water bodies, ultimately contributing to improved public health strategies.

## Materials and methods

### Sampling sites

**Fig 1:**
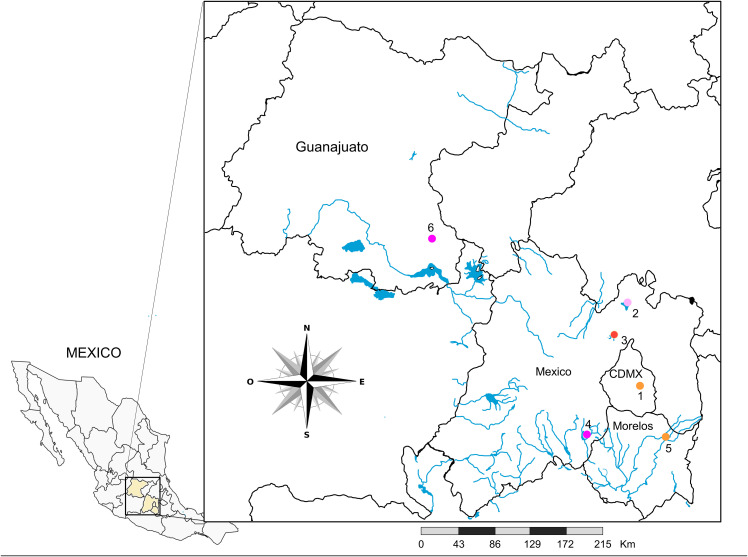
Locations of collection sites. Sampling sites across urban and semi-urban areas in Mexico City (CDMX) and the states of Mexico, Morelos, and Guanajuato. The six water bodies include: (1) Xochimilco Channel (dark purple dot), (2) Zumpango Lagoon (light pink dot), (3) Guadalupe Lake (bright orange dot), (4) Tenancingo Lagoon (red dot), (5) Cocoyoc Lagoon (light purple dot), and (6) Laja River (pink dot). The map was obtained and modified from the INEGI website (https://www.inegi.org.mx/app/mapas/).

Water samples were collected from six water bodies (Fig 1) between January 2018 and September 2018: Xochimilco Channel (XO-CH) in Mexico City (CDMX); Zumpango Lagoon (ZUM-LGN), Guadalupe Lake (GUA-LK) and Tenancingo Lagoon (TGO-LGN) in the State of Mexico (EDOMEX); Cocoyoc Lagoon (CO-LGN) in the State of Morelos (MOR); and Laja River (LA-R) in the State of Guanajuato (GTO). Specific dates, temperatures, pH levels, coordinates, vegetation type, and primary local economic sectors are displayed in Table 1.

**Table 1:** Geographic and physical characteristics of collection sites.

Collection Site and Municipality	Date	T (°C)	pH	GPS Coordinates	Vegetation Type/ Economic Sectors
1. XO-CH, CDMX	03/2018	17.8	7.5	19°15’08.3“N 99°05’34.9”W	Mixed forest, scrubland, grassland/ Agricultural, forestry, conservation, trade.
2. ZUM-LGN, EDOMEX	03/2018	12.6	7.2	19°47’04.0“N 99°08’35.2”W	Xerophytic scrub, Crassicaule, grassland, halophytic grassland/ Agricultural, forestry, fishing.
3. GUA-LK, EDOMEX	01/2018	15.3	7.6	19°37’56.6“N 99°15’31.4”W	Mixed forest, induced vegetation, grassland/ Agricultural, ecotourism, manufacturing.
4. TGO-LGN, EDOMEX	01/2018	19	7	18°57’07.1“N 99°34’06.9”W	Cloud forest, temperate forest, semi-humid forest, xeric scrub/ Agricultural, manufacturing, construction.
5. CO-LGN, MOR	01/2018	21	7	18°52’44.5“N 98°59’00.3”W	Deciduous forest/ Agricultural, tourism, manufacturing.
6. LA-R, GTO	02/2018	17.4	7.8	20°28’56.7“N 100°48’03.4”W	Scrubland, grassland/ Agricultural, tourism, manufacturing, forestry, mining.

T: Temperature; Date: Date of collection; Collection sites are shown in Fig 1. XO-CH, CDMX: Xochimilco Channel, Mexico City; ZUM-LK, EDOMEX: Zumpango Lake, State of Mexico; GUA-LK, EDO.MEX: Guadalupe Lake, State of Mexico; TGO-LGN, EDO.MEX: Tenancingo Lagoon, State of Mexico; CO-LGN, MOR: Cocoyoc Lagoon, Morelos; LA-R, GTO, Laja River, Celaya, Guanajuato.

These natural water bodies were chosen for their ecological qualities, such as land use, possible contamination with pathogenic microorganisms, and environmental contamination due to human activities. Additionally, at these sites, the network of drainage systems, human population growth, and directional flow of subsurface water leads to known pollution. The anthropogenic environmental impact in the vicinity of the collection sites included excessive agricultural operations, fishing, forestry, trade, tourism, and recreation. Samples from each collection site were taken using sterilized 600 ml high-density polyethylene bottles. Samples were transported to the laboratory at 0 °C and either processed the day after collection or stored at −70 °C until analyzed. Sterile distilled water was included as a negative control and processed under the same conditions as the environmental water samples. The complete absence of detectable peptides or proteins in this control was verified by Coomassie-stained SDS–PAGE (Sodium dodecyl sulfate polyacrylamide gel electrophoresis) analysis (S1 Fig).

### Permits and regulatory compliance

This study did not require specific permits. Field activities involved only non-invasive water sampling and did not include the collection, capture, or handling of wildlife, nor access to federally protected areas. In accordance with NOM-059-SEMARNAT-2010 and NOM-126-SEMARNAT-2000, public water sampling without the extraction of flora and/or fauna does not require government authorization. All procedures complied with relevant national and local regulations.

### Protein extraction

Total protein was extracted following procedure described by Ogunseitan, (1993) [21] with a limited number of modifications. From 600 ml water samples, microorganisms and other particulate matter were collected from the aqueous phase by centrifugation at 15,344 × g (11,500 rpm) for 50 min. The supernatant was discarded after determining the absence of protein, and the pellet was manually homogenized using a magnetic stirrer in 1 ml lysis buffer (50 mM Tris HCl, Triton X-100 1%) to extract the protein. Then, the solution was centrifuged at 15,521 × g (12,000 rpm) for 30 min, and the pellet was suspended in 100 µl of lysis buffer once again. This suspension was then centrifuged again at 15,521 x g (12,000 rpm) for 30 min. Both supernatants were then combined, and four volumes of cold (−20 °C) acetone were added overnight to precipitate proteins.

After centrifugation at 10,274 × g (9,500 rpm) for 15 min, the recovered pellet was suspended in 400 µl of cold (−20 °C) acetone (and kept at −20 °C overnight). Then, the suspension was centrifuged at 10,274 × g (9,500 rpm) for 30 min, with the pellet being recovered and allowed to dry at room temperature. Finally, the dried pellet containing the protein was manually suspended using a micropipette in 20 µl of urea buffer (100 mM Tris-HCl, 6 M urea) and centrifuged at 10,274 × g for 30 min. An aliquot of 1 µl was taken to measure protein concentration. The recovered supernatant was kept at −20 °C until use. The negative control was also included and processed under the same conditions as the environmental water samples. The 12% SDS–PAGE was performed according to Brunelle and Green (2014) [22]. The Page Ruler protein ladder used was obtained from Thermo Fisher Scientific (Cat. No. 26619). The complete absence of detectable peptides or proteins in this control was verified by Coomassie-stained SDS–PAGE analysis (S1 Fig). Samples were prepared for proteomic analysis using standard procedures, including trypsin digestion [23]. Afterward, they were purified with ZipTip^®^ Pipette Tips (Millipore Sigma, Burlington, MA), and an aliquot of 15 μl was taken to the Proteomics Unit of the Central Laboratories, Center for Research and Advanced Studies of the National Polytechnic Institute for further analyses.

### Mass Spectrometry Analysis (LC-ESI-HDMSE)

Protein-peptide solutions that were obtained were analyzed in a mass spectrometer with electrospray ionization (ESI) and ion mobility separation (IMS), Synapt G2-Si (Waters Corporation, Milford, MA), using a data-independent acquisition (DIA) approach in HDMSE mode. All generated files containing MS and MS/MS spectra were processed using Protein Pilot and ProteinLynx Global SERVER (Waters Corporation, Milford, MA) and compared against a Mascot knowledgebase, with one missed cleavage site by trypsin permitted (All raw data tables are available in the Harvard Dataverse repository: https://doi.org/10.7910/DVN/MIE2N5). Data obtained were manually searched against the National Center for Biotechnology Information (NCBI) protein database without a taxonomic filter. Taxonomic analysis of detected organisms was determined in accordance with NCBI (https://www.ncbi.nlm.nih.gov/Taxonomy/Browser/wwwtax.cgi) and the Baltimore Classification System [24,25]. Identified peptides ranged in length from eight to 44 amino acids and matched unique genera or species with 100% accuracy. After data were reviewed, a selection of peptides meeting any of the following criteria were reported: (i) sequences observed two or more times in the set of samples, ii) sequences of two or more peptides from the same protein [26], iii) peptide sequences from the same organism, or iv) identified proteins by ProteinPilot Software.

### Data analysis

The richness of families and genera was determined for each sampled water body and represented in donut charts, categorized by taxonomic groups containing pathogenic and non-pathogenic organisms (Fungi, Protozoa, Bacteria and viruses). The intersections of families and genera across sites were visualized using ChiPlot (https://www.chiplot.online/). Subsequently, the relative abundance of each family (pi), which is the proportional representation of a family within a community or sample was calculated using the following formula:


$$\text{pi}=\left(\frac{\text{ni}}{\text{N}}\right)*\text{ 100}$$

Where “ni” is the number of individuals of the same family and “N” is the total number of individuals for all families.

Finally, diversity indices, including Simpson and Shannon indices, as well as heat maps and dendrograms were calculated and generated using PAST 4.03. The Jaccard similarity algorithm was applied hierarchically with 1,000 bootstrap replicates. Each species, identified based on distinct peptides (corresponding to each genus), was treated as the genus frequency. Lastly, similarity in family composition was assessed using multidimensional scaling (MDS), also using PAST 4.03. All data and supplementary tables are available from the Harvard Dataverse repository (https://doi.org/10.7910/DVN/MIE2N5). In total, 10,500 proteins were identified, with 555 peptides showing 100% sequence identity to specific proteins based on NCBI’s BLASTp analysis, some of which corresponded to distinct isoforms of the same protein (S4 Table is available at https://doi.org/10.7910/DVN/MIE2N5)

## Results

### Proteomic identification and taxonomic classification

Protein extracts were analyzed using mass spectrometry to identify organisms in the sampled water bodies that samples were collected from. Our analysis included peptide proteins displaying a 100% identity match with Plantae, Animalia, Archaea, Fungi, Protozoa, Bacteria, Chromistan, and viruses, (S1-S5 Tables at https://doi.org/10.7910/DVN/MIE2N5). After peptides were detected though MS/MS spectra analysis against the Mascot database, which yielded 10,500 proteins, 555 peptides were identified by protein, using NCBI’s BLASTp (All detailed proteomic data and supporting information (S1–S7 Tables) are publicly available in the Harvard Dataverse repository: https://doi.org/10.7910/DVN/MIE2N5). Several peptides were identified as belonging to different isoforms of the same protein (S4 Table is available at https://doi.org/10.7910/DVN/MIE2N5).

### Comparative analysis of richness across water bodies

**Fig 2:**
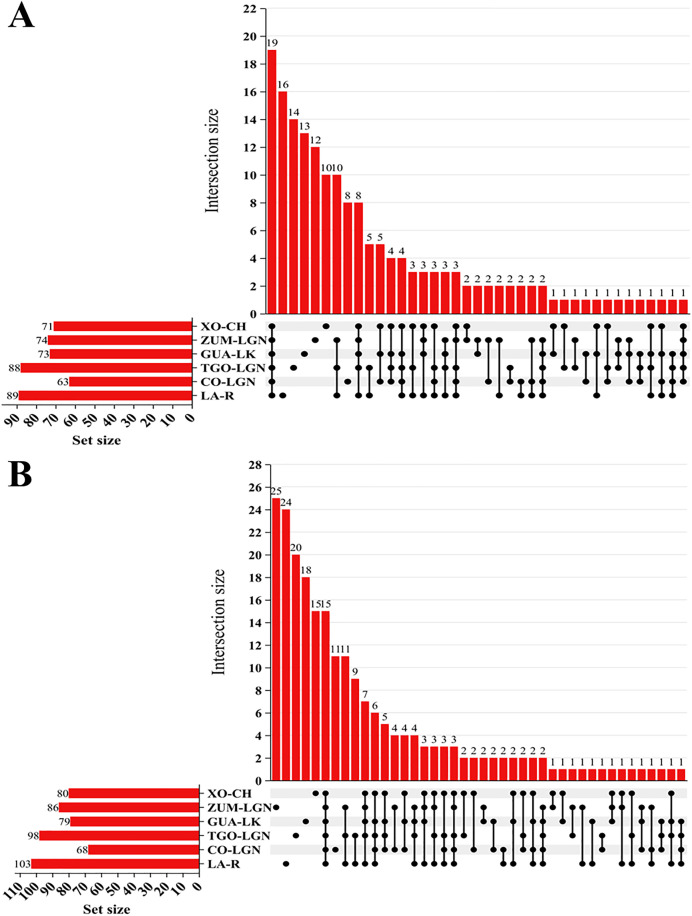
UpSet plot illustrating shared families (A) and genera (B) identified among sampled water bodies. In graphs A (Families) and B (Genera), the number of shared taxa at each site is shown as vertical bars on the right side of the image. Black dots indicate sites that share the number of taxa represented by the vertical bars. The total number of taxa present at each site is also represented by horizontal bars on the left side.

Fig 2 illustrates the total number of families (Fig 2A) and genera (Fig 2B) identified at each sampling site, and shared between water bodies. A total of 172 families and 223 genera were identified across all sites.

Site XO-CH contained 71 families and 80 genera, including 10 families and 15 genera that were unique to the site; ZUM-LGN contained 74 families and 86 genera, including 12 families and 25 genera that were unique to this site; GUA-LK exhibited 73 families and 79 genera, including13 families and 18 genera that were unique to the site; TGO-LGN contained 88 families and 98 genera, including14 families and 20 genera that were unique to the site; LA-R contained 89 families and 103 genera, including 16 families and 24 genera that were unique to this site (greatest taxonomic richness); CO-LGN contained 63 families and 68 genera, including 8 families and 11 genera that were specific to the site (lowest taxonomic richness).

Notably, 19 families (Culicidae, Anaplasmataceae, Aspergillaceae, Dehalococcoidaceae, Dictyosteliaceae, Drosophilidae, Entamoebidae, Hexamitidae, Methanosarcinaceae, Mycobacteriaceae, Sphingomonadaceae, Bacillaceae, Plasmodiidae, Pseudomonadaceae, Shewanellaceae, Streptomycetaceae, Treponemataceae, Vibrionaceae, and Dipodascaceae) and 15 genera (Aspergillus, Dehalococcoides, Dictyostelium, Drosophila, Entamoeba, Giardia, Methanococcoides, Oceanobacillus, Plasmodium, Pseudomonas, Sphingopyxis, Streptomyces, Treponema, Vibrio, and Yarrowia) were distributed across the sites. Furthermore, 10 families and 15 genera were exclusive to XO-CH; 12 families and 25 genera to ZUM-LGN; 13 families and 18 genera to GUA-LK; 14 families and 20 genera to TGO-LGN; 8 families and 11 genera to CO-LGN; and 16 families and 24 genera to LA-R (Fig 2 and S6-S7 Tables and at https://doi.org/10.7910/DVN/MIE2N5).

### Pathogenic and Non-Pathogenic Organisms

Organisms were grouped according to being pathogenic or non-pathogenic (S2 Fig) to identify the potential impact of pollutants on human health. Within the Protozoa group five families containing five non-pathogenic genera (S2 A Fig) and seven families containing nine pathogenic genera were detected (S2 B Fig). The pathogenic Protozoa identified across all sampled water bodies were either *Entamoeba*, *Theileria*, or *Plasmodium*. In the fungi group, nine families containing 10 non-pathogenic genera, and 13 families containing 14 pathogenic genera were detected. Proteins belonging to the pathogen *Colletotrichum* were detected at all sampling sites.

The analysis also revealed the presence of 10 viral families and 13 genera, with no single site containing all of them. Notably, the Poxviridae family, was identified in three water bodies, XO-CH, TGO-LGN, and LA-R. Additionally, the most abundant viral family, *Orthoherpesviridae (also known as Herpesviridae*), was identified in XO-CH, GUA-LK and LA-R.

Bacteria exhibited the highest taxonomic richness, relative to other organism types. Fifty-two families comprising 57 non-pathogenic genera were identified, among which, *Dehalococcoides* was present at all sites (S3A Fig). Additionally, 44 families comprising 59 pathogenic genera were identified, some of which were present at more than one single site. The Bacterial genera *Pseudomonas*, *Vibrio*, *Salmonella*, *Anaplasma*, *Sphingobium*, *Symbiobacterium*, *Listeria*, *Streptomyces*, *Mycobacterium*, and *Treponema* were identified at five of the six sites (S3B Fig).

### Relative abundance of identified families

**Fig 3:**
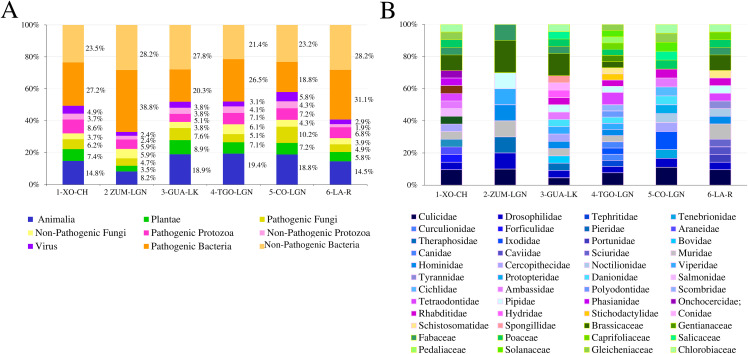
The abundance of families in sampled water bodies. **(A)** The abundance of families for each taxonomic group, represented by the percentage of Animalia, Plantae, Fungi, Protozoa, viruses, and Bacteria identified at each collection site; **(B)** The abundance of families for the Animalia and Plantae groups detected at each collection site. Xochimilco Channel (1-XO-CH); Zumpango Lagoon (2-ZUM-LGN); Guadalupe Lake (3-GUA-LK); Tenancingo Lagoon (4-TNG-LGN); Cocoyoc Lagoon (5-CO-LGN); and Laja River (6-LA-R).

To determine the most representative taxonomic group of each sampled water body, relative abundance was calculated (Fig 3A). The Animalia group (Kingdom) was the second most abundant in all of the sampled water bodies, showing the highest relative abundance at TGN-LGN (19.3%). The plantae group (Kingdom) exhibited its greatest abundance at GUA-LK (8.9%). The greatest percentage of pathogenic and non-pathogenic fungi was at CO-LGN (10.2%) and TGO-LGN (6.1%) respectively. The greatest percentage of pathogenic and non-pathogenic Protozoa was at XO-CH (8.6%) and CO-LGN (4.3%), respectively. The greatest relative abundance of viruses was identified at COL-GN (5.8%). Across all sites, Bacteria were the most relatively abundant taxonomic group. The greatest proportion of pathogenic Bacteria was at ZUM-LGN (38.8%) while the greatest proportion of non-pathogenic Bacteria was at ZUM-LGN (28.2%) and LA-R (28.2%) (Fig 3A).

**Fig 4:**
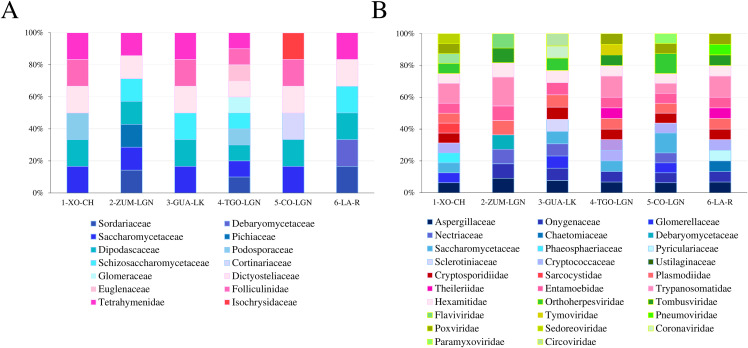
The abundance of families of Protozoa, Fungi, and viruses in sampled water bodies. (A) The relative abundance of families that include non-pathogenic genera of protozoa and fungi. **(B)** The relative abundance of families that include pathogenic genera of Protozoa, Fungi, and viruses. Xochimilco Channel (1-XO-CH); Zumpango Lagoon (2-ZUM-LGN); Guadalupe Lake (3-GUA-LK); Tenancingo Lagoon (4-TNG-LGN); Cocoyoc Lagoon (5-CO-LGN); and Laja River (6-LA-R).

Next, we analyzed the abundance of each family by taxonomic group across all water bodies, finding the most abundant family in each taxonomic group, and at which sampling site this occurred: Culicidae (11.1%) found at CO-LGN and Ixodidae (11.1%) found at CO-LGN, representing the animalia group; Brassicaceae (9.5%) found at ZUM-LGN (Fig 3B), representing the plantae group; there was no difference in abundance among families (Fig 4A) in non-pathogenic Fungi and Protozoa groups; Saccharomycetacea (12.5%) found at CO-LGN, and Trypanosomatidae (18.2%) found at ZUM-LGN (Fig 4B) representing pathogenic Fungi and Protozoa groups, respectively; and Orthoherpesviridae (12.5%) found at CO-LGN, representing the virus group (Fig 4B).

**Fig 5:**
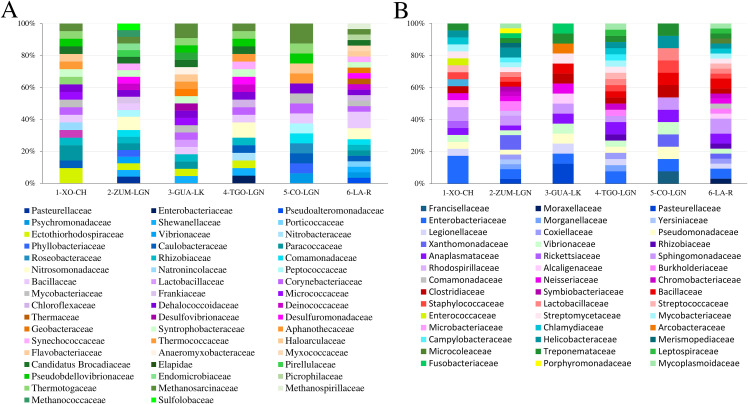
The abundance of families of bacteria in sampled water bodies. **(A)** The relative abundance of families that include non-pathogenic genera. **(B)** The relative abundance of families that include pathogenic genera. Xochimilco Channel (1-XO-CH); Zumpango Lagoon (2-ZUM-LGN); Guadalupe Lake (3-GUA-LK); Tenancingo Lagoon (4-TNG-LGN); Cocoyoc Lagoon (5-CO-LGN); Laja River (6-LA-R).

Due to the importance of non-pathogenic (Fig 5A) and pathogenic bacteria (Fig 5B) we determined abundance in each subgroup (Fig 5; S1 Table, https://doi.org/10.7910/DVN/MIE2N5). The most abundant non-pathogenic bacteria family, was Methanosarcinaceae, found at its greatest abundance at CO-LGN (12.5%), followed by Bacillaceae found at its greatest abundance at LA-R (10.3%) (similar abundance patterns were observed at the other collection sites). Enterobacteriaceae and Pasteurellaceae, belonging to pathogenic Bacteria, were detected in all sampled water bodies. Among the pathogenic Bacterial families, Enterobacteriaceae was the most abundant, exhibiting its highest relative abundance at site XO-CH (18.2%). This was followed by Pasteurellaceae, which reached its greatest abundance at GUA-LK (12.5%) (Fig 5B).

### Genera frequency and distribution across collection sites

**Fig 6:**
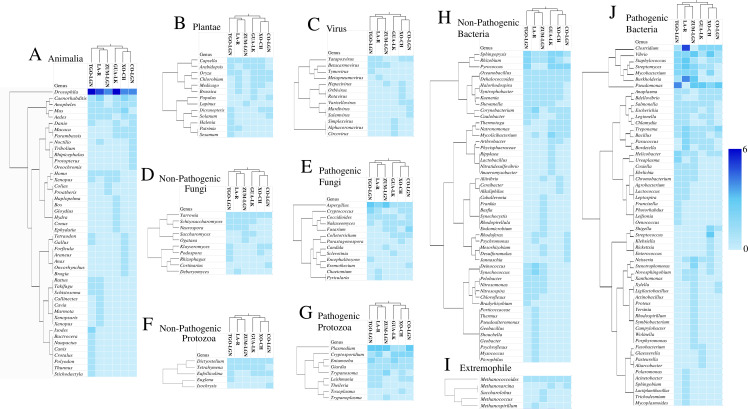
Heat maps of the relative abundance of Animalia, Plantae, Fungi, Protozoa, and Bacteria genera and virus across collection sites: (1). for Xochimilco Channel (XO-CH); (2). for Zumpango Lagoon (ZUM-LGN); (3). for Guadalupe Lake (GUA-LK); (4). for Tenancigo Lagoon (TGO-LGN); (5). for Cocoyoc Lagoon (CO-LGN); (6). for Laja River (LA-R). The heat map shows: **A)** Animalia, **B)** Plantae, **C)** Virus, **D)** Non-Pathogenic Fungi, **E)** Pathogenic Fungi, **F)** Non-Pathogenic Protozoa, **G)** Pathogenic Protozoa, **H)** Non-Pathogenic Bacteria, **I)** Extremophile, **J)** Pathogenic Bacteria. Darker blue rectangles represent higher abundance, while lighter blue rectangles indicate lower abundance across sites.

Heat maps were generated to visualize the abundance of bacterial, fungal, protozoan, animal genera and viruses across sampling sites (Fig. 6). Overall, ZU-LGN exhibited the highest bacterial genus abundance, with 38.8% pathogenic and 28.2% non-pathogenic Bacteria. The Animalia group was most abundant at TGN-LGN (19.3%). Among non-pathogenic genera of Fungi and Protozoa, the genera Dipodascaceae, Dictyosteliaceae, and Tetrahymenidae were consistently detected at five of the six sites. In contrast, pathogenic fungi and protozoa displayed higher abundance than their non-pathogenic counterparts, which remained relatively uniform across all sites. For instance, the Trypanosomatidae genus was present in four locations (XO-CH, ZU-LGN, TGO-LGN, and LA-R), while GUA-LK showed no predominant pathogenic genus. CO-LGN displayed the greatest abundance of Saccharomycetaceae (12.5%). In Bacteria, pathogenic genera were more abundant overall than non-pathogenic genera. For non-pathogenic bacterial genera, Bacillaceae dominated at LA-R (10.3%), whereas no clear predominance was observed at ZU-LGN, GUA-LK, or TGO-LGN. Among pathogenic bacterial genera, Xanthomonadaceae was most abundant at ZU-LGN (9.1%), whereas Enterobacteriaceae and Anaplasmataceae dominated at TGO-LGN (7.6%), and Sphingomonadaceae was most frequent at LA-R (9.3%). No dominant pathogenic genus was identified in GUA-LK and CO-LGN (Fig 6).

### Diversity indices of families collected

**Fig 7:**
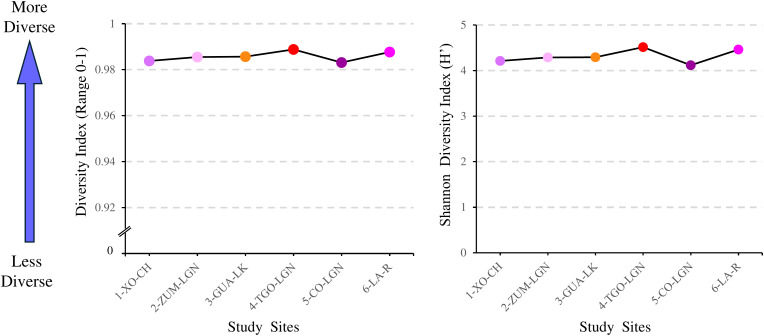
Diversity of families in sampled surface water bodies. **(A)**. The Simpson diversity index for collection sites. **(B)**. The Shannon diversity index at each collection site. Circles represent the diversity index at each site. Xochimilco Channel (1-XO-CH); Zumpango Lagoon (2-ZUM-LGN); Guadalupe Lake (3-GUA-LK); Tenancingo Lagoon (4-TNG-LGN); Cocoyoc Lagoon (5-CO-LGN); Laja River (6-LA-R).

To assess the diversity of each sampled water body at the family level, Simpson and Shannon indices were calculated. The Simpson diversity index indicates the level of diversity in each water body, such as the number of families present at each collection site. High diversity was recorded across all sites (Fig 7A). Similarly, Shannon diversity index values were also high at all sampling sites, indicating high diversity (Fig 7B). Furthermore, the Shannon index indicated communities with greater richness and a more even distribution of families.

## Discussion

Anthropogenic activities contribute to rapid eutrophication of urban water bodies and semi-urban water bodies, altering biodiversity among native organisms and creating harmful imbalances [3,27–31]. While proteomic analyses have been limited in aquatic environments, they are critical for detecting and identifying both beneficial and harmful microorganisms [32], also, conducting such studies allows us to evaluate the effects of pollution, industrial activities, climate change, and other human actions on microbial biodiversity-many of which can significantly impact ecological sustainability and human health. The application of proteomics to the analysis of aquatic pollution, through the search for protein biomarkers, represents a valuable tool for the early detection of possible contaminant exposure and the early evaluation of contamination on microbial communities, several studies support the relevance of proteomic analyses in aquatic environments; for example, López-Pedrouso et al. (2020) highlighted that applying proteomics to the study of aquatic pollution through the identification of protein biomarkers is a valuable tool for the early detection of contaminant exposure and for assessing the effects on aquatic organisms. Similarly, Gajahin Gamage et al. (2022) noted that ecotoxicoproteomics is a rapidly expanding field that enhances the monitoring and analysis of aquatic ecosystems while elucidating the molecular mechanisms underlying biological processes in these environments. Such studies are also useful for identifying bacterial contaminants in aquaculture systems (Çağatay, 2024) [33–36].

### Findings on pathogenic microbes

Worldwide, diseases transmitted by contaminated water cause approximately 2 million deaths per year and have an economic cost of approximately 12 billion US dollars [37]. Contaminated water can transmit a variety of infectious agents, including those that are Bacteria, fungi, protozoa, and viruses, [38]. In this study, major pathogenic microbial communities found in urban and semi-urban water bodies in central Mexico were as follows (S1-S3 Tables and at https://doi.org/10.7910/DVN/MIE2N5): (a) fungi (*Aspergillus fumigatus*, *Coccidioides immitis* and *Candida albicans*); (b) protozoan parasites (*Trypanosoma cruzi*, *Entamoeba histolytica* and *Giardia intestinalis*); (b) pathogenic bacteria (*Escherichia coli* and *Coxiella burnetiid)*; and (c) viruses (*Pigeon circovirus*, *Avian metapneumovirus, Herpesvirus, and Tymovirus chayotis)*. These results indicate a high likelihood that many pediatric cases of diarrheal and enteric disease may cause by in the local water supply. Identifying protein peptides critical for viral replication, release, and pathogenesis [39] is crucial for recognizing microorganisms responsible for human diseases ranging from mild respiratory infections (“colds”) to severe acute respiratory syndrome [40]. Additionally, such knowledge is important for predicting disease outbreaks within communities [41–43].

Water bodies located in semi-urban areas (TGO-LGN and LA-R) showed the greatest biotic diversity. In both areas, *Leptospira* was detected, a genus of zoonotic bacteria associated with leptospirosis, a disease that can become fatal if it progresses to Weil’s disease or severe pulmonary hemorrhagic syndrome. *Leptospira* is globally distributed and generally exhibits a significant interface points with humans and animals. In 2024, Mexico reported 500 cases of leptospirosis during epidemiological week 51 [44]. Additionally, our study identified *Schistosoma*, the causative agent of schistosomiasis [45]. Although, cases of schistosomiasis have been reported in Mexico, thus far reported cases have only occurred in individuals arriving from other countries, not present in the environment [46,47].

In this study, we identified 95 genera of pathogenic microorganisms, of which only four were found across all sampled water bodies: one fungus (*Colletotrichum*), and three protozoa (*Plasmodium, Theileria and Entamoeba*). Moreover, among the pathogens identified in at least five water bodies, there were 11 shared genera, comprising one protozoan (*Giardia*), one fungal (*Coccidioides*) and nine bacterial genera (*Pseudomonas*, *Vibrio*, *Salmonella*, *Anaplasma*, *Symbiobacterium*, *Listeria*, *Streptomyces*, *Mycobacterium*, *and Treponema*). In previous studies in Mexico, *Pseudomonas* have been identified in Chapultepec and Alameda Oriente Lakes (Mexico City) [27]. Furthermore, *Giardia* has been detected in Chalco Lake (Mexico State) [28], and *Vibrio* in Xochimilco Lake (Mexico City), Xaltocan Lake (Mexico State), and Santa Cruz Canal (Mexico City) [48]. These findings are in alignment with our study. In fact, the protozoans (*Cryptosporidium*, *Naegleria fowleri*, and *Giardia*) and bacteria (*Salmonella typhimurium*, *Vibrio cholerae*, *Pseudomonas spp*., *Legionella*, *Escherichia coli* O157:H7, and *Campylobacter jejuni*, *Legionella spp*., *Leptospira spp, E. coli* and *Enterococcus*) identified in this study have caused large outbreaks in Mexico [14,49–55].

### Findings on non-pathogenic microbes

A number of non-pathogenic prokaryotic communities that degrade pollutants and metallic compounds previously reported to be present in wastewater and sewage (*Pyrococcus abyss*i, *Rhodoferax*, *Polaromonas*, and *Chromobacterium, Rhodoferax*, and *Chromobacterium violaceum*), were identified in this study (S2 Fig). Detecting these microorganisms is crucial for assessing water quality and understanding whether their bioremediation capabilities can mitigate risks to human health, protect aquatic ecosystems, and maintain biodiversity. Evidence from similar proteomic studies has identified proteins involved in the activity and metabolism [56] of the archaeon tungsten-dependent *Pyrococcus abyssi,* belonging to the *Thermococcaceae* family, as mediated by sulfur [57]. Proteins from *Rhodoferax, Polaromonas*, and *Chromobacterium* were detected after biostimulation with vegetable oil contamination. [58]. They have also discovered proteins involved in sulfate-reduction from the bacterium *Rhodoferax* [59]. Interestingly, *Dehalococcoides,* which has been used for dechlorination in sites contaminated with chlorinated ethene [60] and *Methanococcoides,* which can be used for biofuel production [61] were also identified.

Non-pathogenic protozoa, chromista, and yeast were also discovered in our analysis, which reflects the contribution of soil and plant debris to water bodies. Our study, along with previous research, has described ribosome peptides from the plastid of *Euglena gracilis* and a peptide from the *Isochrysis galbana* chloroplast that can grow in eutrophicate water bodies [62–64]. Within plantae, peptide sequences for seven families of *Magnoliopsida* [65], which corresponds with Mexico’s wide angiosperm diversity, were identified. At sites XO-CH and CO-LGN, we detected the peptide of the 2S storage protein, which has been reported to be a major allergen in seeds [66]. The most abundant plant family identified in all sampled water bodies except CO-LGN was Brassicaceae (the mustard family). The peptides identified in this family correspond to wild species such as *Arabidopsis thaliana*, *Capsella grandiflora*, and the agricultural species *Brassica juncea* and *Raphanus sativus*.

While non-pathogenic microorganisms play a vital role in bioremediation, environmental conservation, and wastewater treatment (are not associated with traditional human diseases), their proliferation in large quantities can negatively impact health — leading to issues such as gastrointestinal diseases and opportunistic infections [11]. Within the Animalia group, peptides of various organisms belonging to insects, nematodes, arachnids, amphibians, cnidarians, gastropods, reptiles, fish, birds, and mammals were identified in all body waters. An example is the secreted histamine binding protein from the Ixodidae (hard ticks) family identified in CO-LGN and TGO-LGN. Ixodidae are relevant to human and animal health, as these ectoparasites transmit a broad range of pathogens, leading to a spectrum of tick-borne disease [67]. The Culicidae (mosquito) family was the most abundant in all sites except for GUA-LK. Like Ixodidae, Culicidae are often vectors of disease that affect human and domestic animal health, causes diseases such malaria, filariasis, encephalitis, yellow fever, chikungunya, zika, and dengue fever [68]. Notably, 125,160 cases of dengue fever alone were detected in Mexico in 2024 [44]. It is important to note that the detection of Culicidae and Ixodidae peptides in the analyzed water samples provides molecular evidence of proteins associated with these vectors. Such peptides can be considered molecular proxies of vector presence in aquatic environments. Previous studies have reported that the detection of these biomarkers functions as environmental evidence of community presence and biodiversity [69].

A point to worth underscoring is that all of the sampled water bodies exhibited high biotic diversity. When comparing the six water bodies, ZUM-LGN had the highest percentage of pathogenic bacteria (38%) and the lowest percentages of both animal (8.2%) and plant (3.5%) material. Sites GUA-LK and CO-LGN showed higher percentages of non-pathogenic bacteria compared to pathogenic bacteria, along with greater percentages of animals (≥18.8%) and plants (≥7.2%) material. All analyzed water bodies contained pathogenic microorganisms with the potential to cause human diseases, highlighting the need for continued monitoring to reduce contamination risks, improve water treatment, and prevent outbreaks. The findings from this study establish a robust proteomic baseline for ecological and public health research. Future research should extend this framework by integrating multivariate analyses and physicochemical parameters, including pH, nutrient concentrations, heavy metals, and other chemical contaminants, to achieve a more holistic assessment of water quality and its implications for ecosystem health and disease prevention [70].

Although genomic approaches such as eDNA analysis, 16S/18S rRNA sequencing, and metagenomics have substantially advanced the taxonomic characterization of aquatic communities, they still offer only a partial view of ecosystem dynamics because they provide limited information on the functional or metabolic states of organisms. Genomic analyses based on eDNA have transformed biodiversity monitoring; however, several methodological constraints persist. For instance, obtaining sufficient quantities of high-quality DNA can be difficult, as eDNA degrades rapidly and is generally less stable than proteins, which can compromise the reliability of the biological signal. Additionally, the high cost of next-generation sequencing continues to limit its use in large-scale or long-term monitoring programs. Technical artifacts, including PCR amplification biases and sequencing errors, may further introduce false sequence variants, potentially leading to an overestimation of biodiversity within the sampled ecosystem [71]. Collectively, these limitations underscore the need for complementary approaches—such as proteomics—that can provide direct functional insights and enhance the resolution of ecosystem assessments. Proteomic strategies offer a complementary perspective by detecting only the proteins actively expressed at the time of sampling, reflective of only viable and metabolically active biota rather than residual or historical DNA. Unlike amplicon-based sequencing, proteomic analysis can reveal biochemical pathways, stress responses, and trophic interactions linked to environmental conditions. Additionally, proteomics circumvents amplification and primer-bias challenges and can detect taxa for which genomic reference data are scant or completely absent. While not a replacement for DNA-based methods, proteomics adds a functional and ecophysiological dimension that strengthens biomonitoring frameworks when used in combination with existing molecular tools.

Proteomic analysis, however, also presents several limitations, including the complexity of sample preparation and data interpretation arising from the high diversity, dynamic range, and structural heterogeneity of proteins. In addition, the absence of universal protocols, standardized workflows, and baseline criteria makes cross-study comparisons challenging [34]. Variability may also be introduced through differences in instrumentation, technological platforms, and analytical pipelines, which can affect both reproducibility and the depth of protein identification. Despite these challenges, proteomics and genomic approaches should be viewed as complementary rather than competing strategies. Together, they provide a more comprehensive assessment of biological contaminants in water bodies (e.g., lakes, canals) and offer deeper insight into shifts occurring within the native organisms inhabiting these ecological systems.

## Conclusions

In this study the presence of both prokaryotic and eukaryotic communities in urban and semi-urban water bodies were established through proteomic techniques. Microbial contamination was demonstrated through the large variety of pathogens in sampled water bodies, creating a potential environment of harm. Protein biomarkers have been shown to offer a valuable means for the early detection of contaminant exposure and the assessment of their effects on biota in water bodies. Establishing effective biomonitoring protocols, however, presents significant challenges due to the seasonal and spatial variability of communities, as well as the individual diversity of microorganisms in aquatic environments [33].

While the current protein database allows the identification of a wide range of microorganisms—including bacteria, viruses, fungi, protozoa, and even plants and animals—typically only family and genus classifications can be achieved. Accurate species identification requires more specialized analysis, likely using genomic methods.

Unlike genomic analysis, which can provide a clear picture of the presence and viability of specific organisms, proteomics can detect proteins from both live and dead cells, which can lead to potential misinterpretations of environmental health. Meanwhile, DNA extraction and preservation from water bodies can be complex due to susceptibility to degradation, making the use of genomic techniques difficult [72]. Therefore, while proteomics serves as a useful preliminary tool, it may not fully capture the complexities of microbial communities compared to genomic approaches. However, incorporating proteomics into environmental monitoring could significantly strengthen regulatory frameworks such as NOM-230-SSA1–2002, which currently focuses primarily on coliform bacteria. Proteomic analyses offer improved specificity and sensitivity over traditional methods, enabling the identification of proteins associated with a wide range of microorganisms. This approach provides deeper insight into microbial community composition, including potential pathogens that may evade detection by standard assays. Furthermore, proteomic monitoring could support timely responses to pollution events and waterborne diseases, while contributing to the prevention of biodiversity loss among aquatic flora and fauna.

As human activities continue to alter environments and contribute to eutrophication, continuous monitoring of changes to the complex biotic communities of aquatic ecosystems is essential. Ongoing surveillance is vital for regulating water quality, promoting sustainability, and identifying pollution sources that can impact both the environment and public health.

## Supporting information

S1 Fig Coomassie-stained SDS-PAGE analysis of Negative Control Protein for each Water Body Samples. This figure shows the Coomassie-stained SDS-PAGE profile, with the protein negative control derived from distilled water (NC) analyzed alongside the samples collected from the aquatic sites. The NC lanes are displayed in the upper part of the gel and correspond to the controls processed in parallel with samples from the following sites: Xochimilco Channel (XO-CH), Zumpango Lagoon (ZUM-LGN), Guadalupe Lake (GUA-LK), Tenancingo Lagoon (TNG-LGN), Cocoyoc Lagoon (CO-LGN), and Laja River (LA-R). Molecular weight markers (MWM) are displayed on the left side of the image.
(TIF)

S2 Fig Donut chart displaying the richness of non-pathogenic and pathogenic taxa of Protozoa, fungi, and viruses at the family and genus level. (A) Non-pathogenic Protozoa (pink gradients) and fungi groups (blue gradients). (B) Pathogenic Protozoa (pink gradients), fungi (blue gradients), and viruses (each virus indicated using a different color). Capital letters indicate family names, while numbers represent sample collection sites, as follows: 1 for Xochimilco Channel (XO-CH); 2 for Zumpango Lagoon (ZUM-LGN); 3 for Guadalupe Lake (GUA-LK); 4 for Tenancigo Lagoon (TGO-LGN); 5 for Cocoyoc Lagoon (CO-LGN); 6 for Laja River (LA-R). Families containing different genera are represented by different color gradients in the outer circles.
(TIF)

S3 Fig Donut chart displaying the richness of non-pathogenic and pathogenic taxa of Bacteria at the family and genus level. (A) Non-pathogenic Bacteria grouped into 52 families (indicated by blue, purple, pink, green, and orange). (B) Pathogenic Bacteria grouped into 44 families (indicated by blue, purple, green, and orange). Capital letters represent different families, while numbers represent sample collection sites, as follows: 1: Xochimilco Chanel; 2: Zumpango Lake; 3: Guadalupe Lake; 4: Tenancigo Lagoon; 5: Cocoyoc Lagoon;6: Laja River. Families containing different genera are represented by different color gradients in the outer circles. The raw data tables and detailed proteomic analysis results (S1–S7 Tables) have been deposited in the Harvard Dataverse repository and are publicly available at: https://doi.org/10.7910/DVN/MIE2N5.
(TIF)

S1 Table Protein peptides of viruses, archaea, and bacteria identified in collected samples with 100% identity match in the NCBI database. (XLSX)

S2 Table Protein peptides from protozoa, chromista, fungi, and plants identified in the collected samples with 100% identity match in the NCBI database. (XLSX)

S3 Table Protein peptides of Animalia identified in the collected samples with 100% identity match in the NCBI database. (XLSX)

S4 Table Peptides of protein isoforms identified in the collected samples with 100% identity match in the NCBI database. (XLSX)

S5 Table Peptides of conserved proteins from organisms identified in collected samples with 100% identity match in the NCBI database. (XLSX)

S6 Table Unique and co-occurring families identified in sampled water bodies. (XLSX)

S7 Table Unique and co-occurring genera identified in sampled water bodies. (XLSX)
