# Correction: A Preliminary proteomics-based assessment of biotic indicators in Central Mexican water bodies biotic indicators by proteomics in Mexican water bodies

**DOI:** 10.1371/journal.pone.0352092

**Published:** 2026-06-17

**Authors:** Catalina E. Gardella-García, Eduardo Domínguez-de-la-Cruz, Gerardo Pérez-Ramírez, Randy E. David, Juan Enrique Chacón-Hernández, Sandra Cotino-Nájera, María de Lourdes Muñoz

In the title of this article, the phrase “biotic indicators by proteomics in Mexican water bodies” should not be present. The correct title is: A Preliminary proteomics-based assessment of biotic indicators in Central Mexican water bodies. The correct citation is: Gardella-García CE, Domínguez-de-la-Cruz E, Pérez-Ramírez G, David RE, Chacón-Hernández JE, Cotino-Nájera S, et al. (2026) A Preliminary proteomics-based assessment of biotic indicators in Central Mexican water bodies. PLoS One 21(2): e0342705. https://doi.org/10.1371/journal.pone.0342705
