# Supplementary figures and images for: A Preliminary proteomics-based assessment of biotic indicators in Central Mexican water bodies biotic indicators by proteomics in Mexican water bodies
Source: PLoS One. 2026 Feb 26;21(2):e0342705. doi: 10.1371/journal.pone.0342705 (PMC12944710; doi:10.1371/journal.pone.0342705)

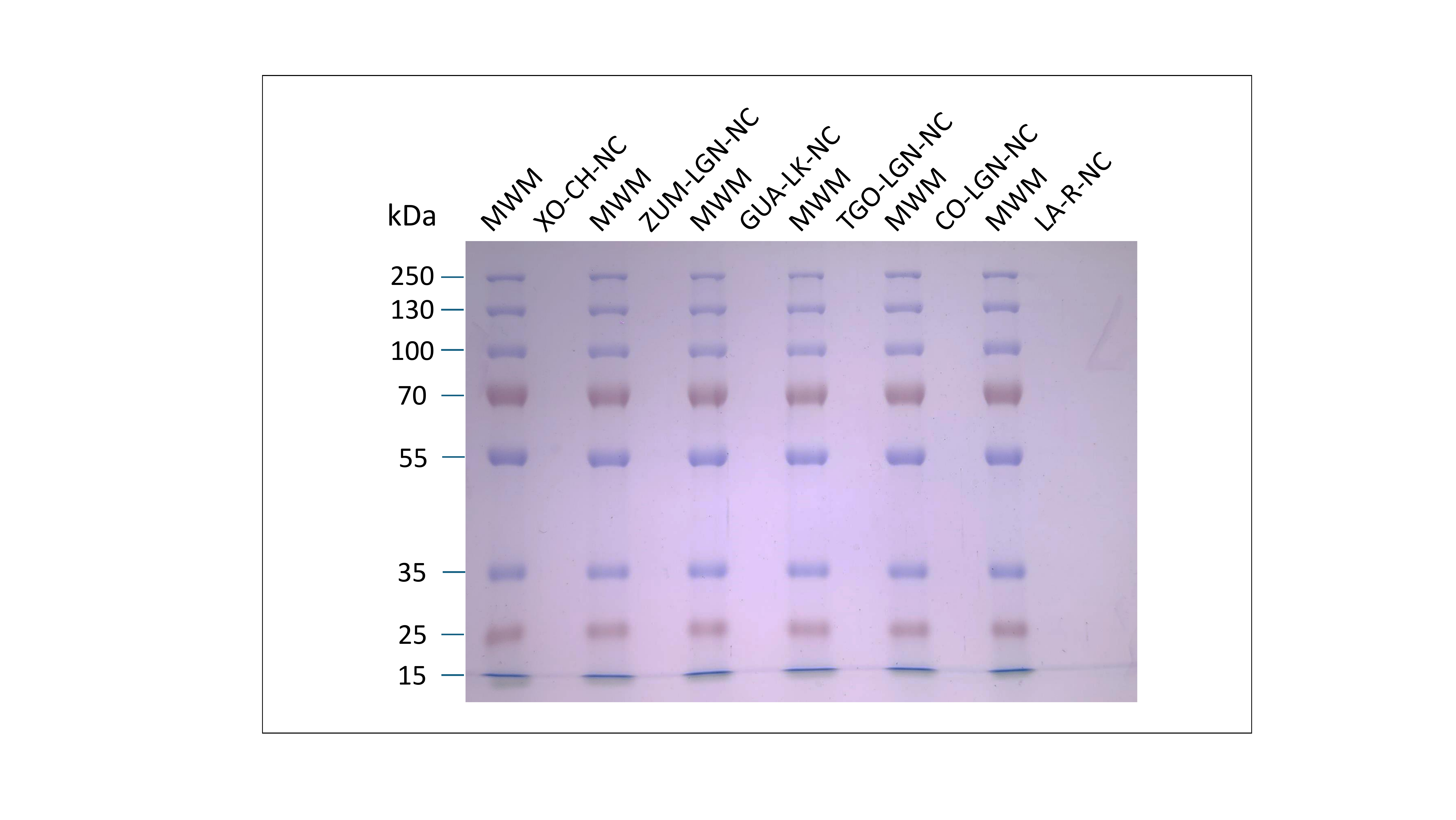

Supplement: S1 Fig — This figure shows the Coomassie-stained SDS-PAGE profile, with the protein negative control derived from distilled water (NC) analyzed alongside the samples collected from the aquatic sites. The NC lanes are displayed in the upper part of the gel and correspond to the controls processed in parallel with samples from the following sites: Xochimilco Channel (XO-CH), Zumpango Lagoon (ZUM-LGN), Guadalupe Lake (GUA-LK), Tenancingo Lagoon (TNG-LGN), Cocoyoc Lagoon (CO-LGN), and Laja River (LA-R). Molecular weight markers (MWM) are displayed on the left side of the image. (TIF) [file pone.0342705.s001.tif]

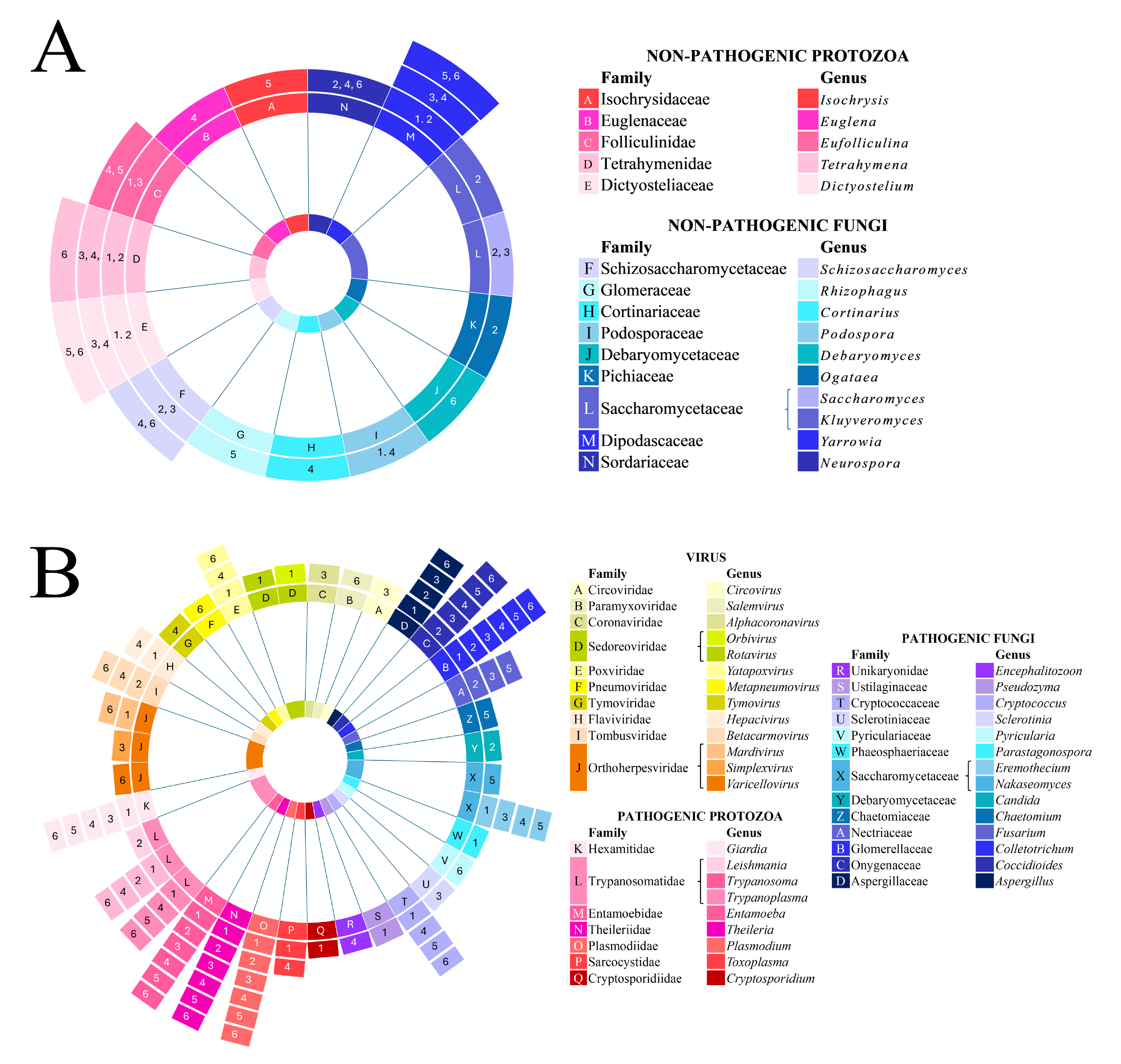

Supplement: S2 Fig — (A) Non-pathogenic Protozoa (pink gradients) and fungi groups (blue gradients). (B) Pathogenic Protozoa (pink gradients), fungi (blue gradients), and viruses (each virus indicated using a different color). Capital letters indicate family names, while numbers represent sample collection sites, as follows: 1 for Xochimilco Channel (XO-CH); 2 for Zumpango Lagoon (ZUM-LGN); 3 for Guadalupe Lake (GUA-LK); 4 for Tenancigo Lagoon (TGO-LGN); 5 for Cocoyoc Lagoon (CO-LGN); 6 for Laja River (LA-R). Families containing different genera are represented by different color gradients in the outer circles. (TIF) [file pone.0342705.s002.tif]

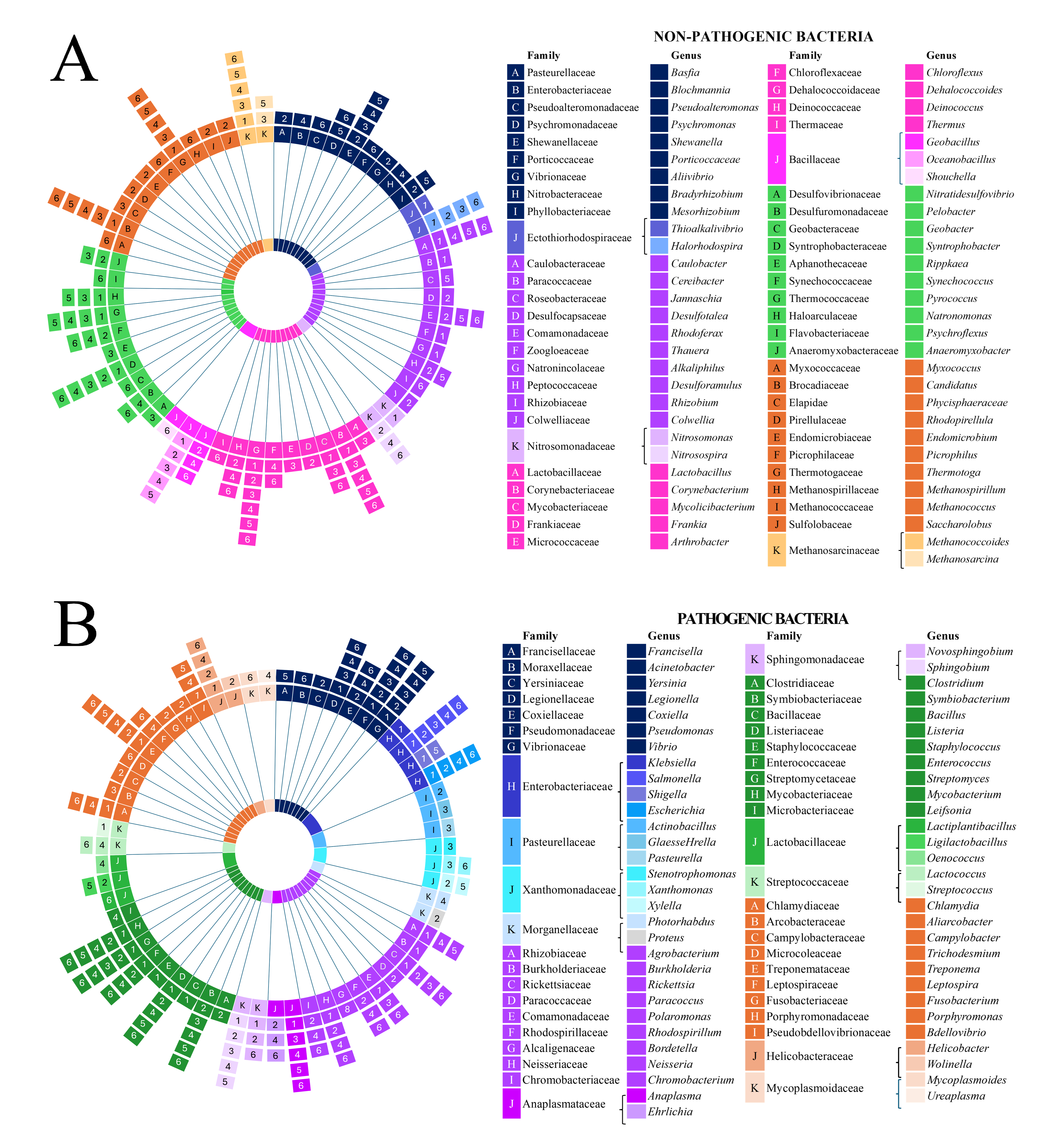

Supplement: S3 Fig — (A) Non-pathogenic Bacteria grouped into 52 families (indicated by blue, purple, pink, green, and orange). (B) Pathogenic Bacteria grouped into 44 families (indicated by blue, purple, green, and orange). Capital letters represent different families, while numbers represent sample collection sites, as follows: 1: Xochimilco Chanel; 2: Zumpango Lake; 3: Guadalupe Lake; 4: Tenancigo Lagoon; 5: Cocoyoc Lagoon;6: Laja River. Families containing different genera are represented by different color gradients in the outer circles. The raw data tables and detailed proteomic analysis results (S1–S7 Tables) have been deposited in the Harvard Dataverse repository and are publicly available at: https://doi.org/10.7910/DVN/MIE2N5. (TIF) [file pone.0342705.s003.tif]
